# Why I Am Not SHY: A Reply to Tononi and Cirelli

**DOI:** 10.1155/2013/394946

**Published:** 2013-02-12

**Authors:** Marcos Gabriel Frank

**Affiliations:** Department of Neuroscience, Perelman School of Medicine University of Pennsylvania, 215 Stemmler Hall, Philadelphia, PA 19104, USA

## Abstract

In a recent article I reviewed an influential theory of sleep function, the “synaptic homeostasis hypothesis (SHY.)” According to SHY, sleep renormalizes synapses that are potentiated during prior wakefulness. I concluded that while SHY is a seminal theory with important implications about sleep function and the brain, its underlying mechanisms are poorly defined. In an accompanying article, the authors of SHY responded at length. Their reply is thoughtful and provocative, but unfortunately many of the points I raised were not accurately represented or addressed. In this brief commentary, I attempt to clarify some points of confusion. I also explain why any theory of sleep function is incomplete without an understanding of the underlying cellular mechanisms.

## 1. Introduction

In their companion article, Tononi and Cirelli argue that I have missed the big picture by conflating questions of sleep function with the underlying mechanisms [1]. As I have discussed elsewhere [2, 3], understanding sleep function is of central importance to biology. Any theory of sleep function must also grapple with universal traits of sleep, some of which were enumerated in their response. There are other theories of sleep function and many of the theoretical arguments made in support of SHY (e.g., a need for offline states, sleep homeostasis, and brain metabolism) apply to them. These are not the issues at hand. The issue is how should scientists evaluate these theories? My position is that this evaluation must always include a discussion of mechanisms, because they cannot be disentangled from functional questions.

 The underlying message from Tononi and Cirelli is that what really matters is the “…the end result” [1, page 4] rather than how you get there. I find this an odd position to take and a backward step in our pursuit of sleep function. The goal of science is to understand how nature works. That includes an empirical pursuit of physical mechanisms. This in part distinguishes science from pure philosophy. Scientists should therefore be skeptical of any theory of sleep function that fails to elucidate the underlying mechanisms that govern the proposed function. In this regard, the proponents of SHY are in an indefensible position when they argue that the mechanisms have “…no bearing on whether the core claim of SHY is true or false” [1, page 3]. They have bearing because if the underlying mechanisms are not sleep dependent then the theory is wrong. Incidentally, the theory is also wrong if the proposed mechanisms do not exist. While it may be true that these mechanisms are complex and perhaps different in different species, that is not a reason to ignore them. On the contrary, that is a reason to explore them.

## 2. Confusion and Conflation

 Tononi and Cirelli suggest that I am simply confused about SHY. Indeed, I do find SHY confusing because the proposed mechanisms (to date) seem inconsistent with what is actually known about synaptic plasticity. Moreover, their imprecise terminology fogs the issue. They claim that they never meant that the potentiation in wakefulness is Hebbian (i.e., some form of LTP), yet it is difficult to draw any other conclusion based on their own words. Their liberal and interchangeable use of the words “long-term potentiation”, “LTP,” or “LTP-like” [4, 5] leaves little wiggle room. This is because “LTP” is not short-hand for “stronger synapse;” it has a precise meaning and commonly refers to Hebbian processes. In their rebuttal, they back peddle from their past specific language regarding the significance of neuromodulator release and the expression of plasticity molecules in SHY. There is, however, little ambiguity in their original description of how these events purportedly promote LTP (or LTP-like events) during wake versus sleep [4, 5]. 

 They further imply that I have conflated accepted meanings of the terms “synaptic scaling” or “synaptic homeostasis” with what they now call “synaptic renormalization.” But I am not the guilty party here. The concepts and terminology of synaptic homeostasis and scaling predate SHY [6] and there is simply no mistaking the broad similarities between synaptic scaling and what is described in SHY. For example, here is a more complete excerpt from Tononi and Cirelli. 

 “*Like activity-dependent synaptic scaling* (emphasis added), however, sleep-dependent downscaling would affect most or all of a neuron's synapses. In this respect, downscaling is conceptually different from long-term depression, which affects select groups of synapses, or depotentiation, which affects only recently potentiated ones” [5, page 54]. 

Therefore, their use of these terms, if in fact they mean something else, is perplexing. The distinctions they make between their version of downscaling and scaling proper are welcome [1], but quite subtle when compared to the overall similarities. For example, synaptic homeostasis is not only manifested at the level of firing rates as implied by Tononi and Cirelli. That is one *outcome* of a homeostatic adjustment of synaptic weights in a network, as measured, for example, by mEPSCs [7, 8]. This point seems moot, since Tononi and Cirelli cite changes in firing rates and mEPSCs as evidence of SHY [9, 10]. 

 In the end, it really does not matter what new name they give to their process (or set of processes) that weakens synapses. The point I was making, and which they missed, is that any form of plasticity (especially if possibly novel) should be examined through the prism of known facts about Hebbian and non-Hebbian plasticity. Case in point: I never stated that the “downscaling” of SHY is *identical *to synaptic scaling as currently understood in the field. I instead argued that since the concepts of scaling figure prominently in SHY, and because scaling is an accepted means of globally adjusting synaptic weight, it is logical to examine the claims of SHY based on what is known about synaptic scaling. I look forward to future efforts to distinguish “synaptic renormalization” from synaptic scaling, but this name change does not inoculate SHY against scrutiny.

## 3. Veracity in Science Is Not by the Pound

 The veracity and validity of a scientific theory is not solely determined by the amount of data piled on one side of a scale. It is also determined by a careful examination of each piece of evidence, pro and con. Upon close examination, threads of supportive findings may unravel, and when contrary evidence is properly considered, an otherwise impressive mass of findings may collapse. A large part of their response is a long catalog of supportive data—some of which I included in my review. However, as I pointed out, careful examination reveals alternative explanations for some of their results. They also largely ignore evidence consistent with net increases in synaptic strength after sleep [6], or findings that question their hypothesized relationship between synaptic potentiation and slow-wave electroencephalographic activity (SWA) [11, 12]. For example, recent work from Chauvette et al. [13] provides compelling evidence that SWA increases synaptic strength *in vivo*. These findings are consistent with my original discussion [6] and completely in opposition to predictions of SHY. When these are also considered the universality of SHY, and its utility as an explanation for why we sleep, becomes questionable.

## 4. A Good Experiment Beats a Good Argument Every Time

 In my original review, I considered biological processes other than sleep that might explain some findings ascribed to SHY. These included the *cumulative* effects of natural patterns in brain temperature (and in mammals, glucocorticoid release) across the 24-hour day or after sleep deprivation. Tononi and Cirelli present a number of arguments against these ideas, but I have a better idea. Why not perform the simple experiments I proposed? For example, why not examine the effects of cooling or warming on Drosophila synaptic proteins (which are altered by changes in temperature as small as 8°C; *not* as they state 20°C)? Why not experimentally clamp corticosterone as done by Mongrain et al. [14] and thus eliminate corticosterone rhythms as a factor in rodent synaptic efficacy? Tononi and Cirelli argue strenuously that SHY is a theory of function, yet they support their argument with cellular and molecular findings that have no demonstrable function. Rather than elaborate upon already dense theoretical arguments about the functional importance of SHY, why not demonstrate it empirically? In science, no argument, no matter how beautifully crafted, trumps a good experiment.

## 5. Concluding Remarks

 In his discussion of sleep theories 50 years ago, Nathaniel Kleitman reminded his readers of the cautionary tale of Ptolemy of Alexandria [15]. Ptolemy was a revered scholar in the 2nd century AD who codified hundreds of years of prior Greek astronomy and mathematics into what came to be known as the Ptolemaic universe. The Ptolemaic universe held that the stars, planets and sun revolved around the earth, embedded in overlapping crystalline spheres. This theory of the Natural world was elegant, mathematical, and even highly predictive of astronomical events. It also was *completely* wrong. The lesson from Ptolemy is that scientific theories are ultimately judged not only by their power to explain, but by the validity of their physical mechanisms. 

 Tononi and Cirelli argue that for SHY to be valid, it does not matter how synapses are weaker after a sleep period, just that they are [1]. But Ptolemy reminds us that this is not enough; scientific truth comes from asking hard questions about how things actually work. I do not question their results, showing for example, changes in evoked responses or mRNAs consistent with SHY. I do question whether these changes are directly caused by sleep. This question arises for two reasons. First, they have not identified how sleep actually alters synaptic strength in *any *species. Second, they have not experimentally excluded the role of other biological processes that coincide with sleep. A final consideration is that if SHY is a scientific hypothesis, then Tononi and Cirelli should propose an experimental outcome that would force a rejection of SHY. The ultimate validity of SHY requires that these points be addressed.
